# Association between irisin and metabolic parameters in nondiabetic, nonobese adults: a meta-analysis

**DOI:** 10.1186/s13098-022-00922-w

**Published:** 2022-10-21

**Authors:** Yan Li, Zhenbin Xu

**Affiliations:** 1 Department of Endocrinology, The Second Affiliated Hospital of Fujian Medical University, Quanzhou, 362000 Fujian China; 2 Department of Orthopaedics, The Second Affiliated Hospital of Fujian Medical University, Quanzhou, 362000 Fujian China

**Keywords:** Metablic syndrome, Irisin, Blood glucose, Insulin resistance, Obesity

## Abstract

**Background:**

Irisin has been proposed to have a beneficial influence on the metabolic status of animals and humans. However, the relationship between circulating irisin levels and the risks of metabolic components in humans remains unclear. In the present meta-analysis, we aimed to evaluate the association between circulating irisin and metabolic parameters in nonobese, nondiabetic adults.

**Methods:**

We searched PubMed, Embase, the Cochrane Library, Web of Science and ClinicalTrial.gov using the main search terms and identified original articles published prior to March 7, 2022. Studies that met our inclusion criteria and reported the association between irisin and metabolic parameters were included in our meta-analysis. We used the Newcastle Ottawa scale to assess the quality of the included studies.

**Results:**

A total of 14 studies (711 subjects) in 11 articles were included for qualitative and quantitative synthesis. The pooled results showed that circulating irisin was positively and significantly correlated with fasting blood glucose (r = 0.159), HOMA-IR (r = 0.217) and waist-to-hip ratio (WHR) (r = 0.168). However, no significant association was detected between irisin levels and other metabolic parameters.

**Conclusions:**

Thus, these findings indicated the possible link between irisin levels and part of the metabolic parameters in apparently metabolically normal individuals. However, the regulation of irisin in metabolism in humans remains to be fully elucidated, and well-designed prospective studies will be needed in the future.

*Trial registration* The review protocol was registered in the International Prospective Register of Systematic Reviews (PROSPERO): CRD42022315269.

**Supplementary Information:**

The online version contains supplementary material available at 10.1186/s13098-022-00922-w.

## Background

Metabolic syndrome (MetS) is a complex pathophysiologic condition characterized by dysregulated glucose homeostasis, abdominal obesity, dyslipidaemia and increased arterial blood pressure [1, 2], and it increases the risk of cardiovascular diseases, stroke and certain types of tumours [3]. The syndrome results mainly from the imbalance of energy intake and consumption, but it is also determined by genetic or epigenetic makeup of individuals and a sedentary or active lifestyle [1]. It is estimated that over a billion people in the world are now suffering from metabolic syndrome, which has become one of the primary causes of morbidity and mortality in the modern world, resulting in financial burdens to society and families worldwide [1]. However, mild metabolic disorders may already take place before any clinical manifestation emerges and is detected by current metabolic biomarkers. Therefore, novel biomarkers are needed in apparently metabolically healthy populations to identify metabolic abnormalities in the early stage.

Irisin, a hormone first described in 2012 by Boström et al. [4], is a thermogenic myokine cleaved from fibronectin type III domain containing protein 5 (FNDC5), which is mainly released by muscle and adipose tissue and secreted upon physical activity [3] and cold exposure [5]. Irisin has been proposed to have a beneficial influence on the metabolic status of animals and humans, especially in inhibiting the accumulation of fat and obesity because it promotes the browning of white adipose tissue [3, 6]. Since the identification of irisin, a hypothesis has been proposed that a beneficial role of exercise in metabolic diseases may be related to irisin, suggesting that it has a potential role in the diagnosis and treatment of metabolic diseases, such as diabetes mellitus and obesity [7, 8]. However, before further exploring the application of exogenous irisin, the rational next step is to clarify the link between endogenous irisin and metabolic parameters as well as the underlying pathophysiological mechanism.

However, the relationship between circulating irisin levels and the risks of metabolic components in humans remains unclear. A cross-sectional study has evaluated the relation of irisin to MetS components in 151 participants and observed positive associations between irisin and various metabolic parameters [9]. Regarding the association between irisin and anthropometric parameters, most evidence shows a positive association between irisin levels and indices of adiposity, including body mass index (BMI), waist circumference, waist-to-hip ratio (WHR) and fat mass [9–15]. However, several studies have not detected any association [16] or reported a negative correlation of irisin with fat mass, BMI and WHR [17]. In patients with type 2 diabetes mellitus and prediabetes, most studies have observed lower circulating irisin levels than controls [17–20], suggesting a downregulated level of irisin under the conditions of impaired glucose tolerance [6]. In contrast, evidence from studies in nondiabetic populations has shown that irisin levels are positively correlated with fasting blood glucose [10, 19, 21], beta cell function [22] and insulin resistance [23]. Similarly, insulin sensitivity has been reported to be negatively associated with irisin [23, 24], except for one study reporting a positive association in a Caucasian population [17]. Regarding lipid metabolism, studies have suggested that the expression of FNDC5 or the administration of irisin promotes lipolysis and inhibits the synthesis of lipids [25–27]. However, data from human research are inconsistent. On the one hand, a positive correlation between irisin levels and an unfavourable lipid profile, such as triglycerides (TGs) [9, 28], total cholesterol (TC), low-density lipoprotein cholesterol (LDL-C) and fasting fatty acids [29, 30], has been reported. Additionally, irisin has been reported to be negatively associated with high-density lipoprotein cholesterol (HDL-C) in patients at high cardiometabolic risk [9, 31]. On the other hand, irisin has been suggested to be inversely associated with total, LDL cholesterol and triglycerides in a Central European general population [32]. However, in several studies, no association of irisin with lipid indices has been reported in healthy and young individuals [33], normal-weight individuals with increased body fat [34] and male subjects with mild hypercholesterolemia [35]. Animal experiments have provided evidence that irisin administration lowers blood pressure [36, 37]. However, few studies have been conducted to investigate the association between irisin levels and blood pressure in humans. Positive correlations have been found between irisin levels and blood pressure in obstructive sleep apnoea patients [38]. Similarly, a study including 24 obese and 63 normal-weight children has found that irisin levels are positively correlated with systolic and diastolic blood pressure [39]. However, a significant relationship between irisin and 24-h blood pressure has not been reported in studies including young hypertensive adults [40].

The inconsistent findings observed across the studies indicate the complexity of the relationship between circulating irisin levels and metabolic components. However, the wide range of BMI and different degrees of glucose homeostasis of subjects across the studies may be sources of clinical heterogeneity. To reduce the influence of obesity or abnormal glucose tolerance on irisin levels, we performed a meta-analysis to evaluate the association between circulating irisin levels and metabolic parameters in nonobese, nondiabetic adults. Thus, the results will be more reasonable in the absence of these two major metabolic components. It should be noted that we did not exclude patients with other metabolic disorders, such as overweight, NAFLD, PCOS or dyslipidaemia.To the best of our knowledge, no systematic review or meta-analysis has been performed on this basis. Thus, the present study provided evidence supporting irisin as a potential biomarker of the risk of metabolic disorders in this population to some extent.

## Material and methods

### Study protocol

The present study was conducted according to the Meta-analysis of Observational Studies in Epidemiology (MOOSE) standards [41]. The review protocol was registered in the International Prospective Register of Systematic Reviews (PROSPERO): CRD42022315269.

### Data sources and search strategy

A systematic literature search of electronic databases, including PubMed, Embase, the Cochrane Library, Web of Science and ClinicalTrial.gov, was conducted by two authors independently using the main search terms and database-specific terms related to “irisin”, “metabolic parameters” and “clinical trials” (see detailed search strategy in Additional file 1). Original articles published prior to March 7, 2022 were identified. The reference lists of relevant reviews and conference abstracts were also screened for potential studies.

### Study selection

The titles and abstracts of articles were screened for potentially eligible studies. The full texts of all eligible articles were then obtained and reviewed by two authors independently to ensure that the articles met the inclusion criteria of the review. Any disagreements about the study selection were resolved by consensus. Studies meeting the following criteria were included: (a) enrolled nonobese, nondiabetic adult subjects; (b) reported the relation of circulating irisin to at least one metabolic parameter, including fasting blood glucose (FBG), homeostasis model assessment-insulin resistance (HOMA-IR), insulin levels, BMI, waist circumference (WC), WHR, TG, TC, LDL-C, HDL-C, systolic blood pressure (SBP) or diastolic blood pressure (DBP), at baseline using Spearman correlation analysis, Pearson correlation analysis or simple linear regression analysis; and (c) designed as a cross-sectional, cohort or case–control study. Studies involving healthy control groups were only included when a BMI less than 30 kg/m^2^ (according to the definition of WHO [42]) could be deduced from the baseline data in the article or was confirmed by the corresponding author, thus overweight participants(25 ≤ BMI < 30 kg/ m^2^) were not excluded from our study. Studies that enrolled patients with impaired glucose regulation or obesity without excluding them from the entire study population when calculating the correlation coefficients were excluded. However, we did not exclude patients with other metabolic disorders. Pregnant women were also excluded considering the complicated and heterogeneous changes during the gestational period.

### Data extraction and quality assessment

Data extraction was conducted independently by two reviewers (L.Y. and X.Z.) using a predesigned standardized data extraction form, which included the first author, year of publication, study location, type of study, sample size, characteristics of participants (sex ratio, age range, BMI range and health status), blood sample, ELISA kits for irisin measurement, analysis methods and correlation or regression coefficients. The corresponding authors of the articles were contacted by email, if necessary, for unpublished information. The methodological quality of each study was assessed according to the Newcastle–Ottawa Scale (NOS) [43], which contains eight items classified into three aspects (selection, comparability and outcome or exposure) [43]. The range of NOS was 0–9 stars, and a study was defined as high-quality when it achieved 7 or more stars. Two researchers (L.Y. and X.Z.) independently assessed the quality of the studies. Any disagreements in any phase were settled through discussion.

### Data analysis

For studies with interventions, only the baseline correlation coefficient was included in the meta-analysis. For studies with more than one eligible group, only the collective correlation coefficients (if provided) were used in our analysis. For studies using Spearman’s method to calculate the correlation coefficients, the correlation coefficients (rs) were converted into approximate Pearson correlation coefficients (r) prior to inclusion in the meta-analysis, considering the significant difference between the two methods [44]. For studies that used simple linear regression to calculate the correlation coefficients, the results were directly applied in the analysis. Fisher's z transformation of correlation coefficients, the variance of z and standard error were calculated using the following formulas before obtaining the summary effect size (r) [45]:

$$Fisher'z = 0.5 \times In\left[ {\left( {1 + r} \right)/\left( {1 - r} \right)} \right]$$

$${V}_{z}=\frac{1}{n-3}$$

$${S}_{E}=\sqrt{{V}_{z}}$$

$$Summary r=({e}^{2Z}-1)/({e}^{2Z}+1)$$

All metabolic parameters assessed were considered as continuous variables. The pooled effect estimates were calculated using a random-effect model. The inverse variance method was applied to estimate the weight of each study. Statistical heterogeneity was tested using the chi-squared test, and I^2^ with a value greater than 50% indicated substantial heterogeneity across studies. Additionally, sensitivity analyses were performed to assess the robustness of the results using the leave-one-out method. Furthermore, subgroup meta-analyses were conducted to explore the heterogeneity and to evaluate the impact of specific factors on the outcome estimates. Publication bias was assessed with funnel plot analysis and Egger's regression asymmetry test. All statistical analyses were performed using RevMan 5.3.5 (The Nordic Cochrane Centre, the Cochrane Collaboration, 2014) or Stata Software 16.0 (College Station, TX, 77845, USA).

## Results

### Search results

**Fig. 1:**
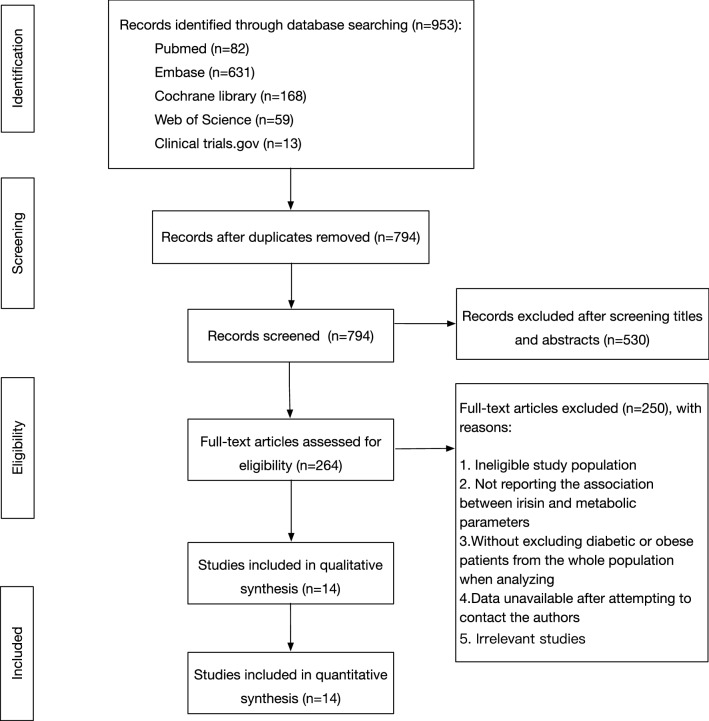
Flowchart of the selection procedure of the included studies

A total of 953 studies were identified through database searching. After the removal of duplicates, 794 unique records were screened by reading the titles and abstracts. The full texts of 264 articles were then retrieved for detailed assessment. Studies with ineligible study subjects or that did not report the outcome of interest were excluded. Studies were also excluded when they enrolled diabetic or obese subjects without excluding them from the entire population when calculating the correlation coefficients. Studies using multivariate linear regression to calculate the correlation between irisin and metabolic parameters were also excluded, considering the complexity of various confounders and models in different studies. The corresponding authors of articles in which data were inadequate for our meta-analysis were contacted by email. Overall, 14 studies in 11 articles were included for qualitative and quantitative synthesis. The detailed process of study selection is illustrated in Fig. 1.

### Study characteristics

The characteristics of the included studies are summarized in Table 1. The selected studies were published between 2013 and 2022 with five cross-sectional studies and nine case–control studies. Regarding the location of the studies, four studies were conducted in Europe, five studies were conducted in Asia, four studies were conducted in Africa and one study was conducted in Australia. The sample sizes ranged from 26 to 122 with a median of 45. Of the 711 participants in the included articles, 410 (57.7%) were females. Two studies recruited only male subjects, and 6 studies recruited only female subjects. Most studies (n = 8) recruited healthy subjects or included healthy control groups, except for one study involving nonalcoholic fatty liver disease patients, three studies involving polycystic ovary syndrome (PCOS) patients, one study including mild hypercholesterolemia and one study including normal weight individuals with increased body fat (> 30%). Nine studies used serum blood samples of irisin, and five studies used plasma irisin. For the ELISA kits used to measure circulating irisin levels, three studies used Phoenix Pharmaceuticals, six studies used BioVendor and five studies used other kits. Some studies strictly controlled for potential confounders, such as physical activity [33, 46, 47] and daily energy or macronutrient intake of subjects [33, 46, 47] before examination, considering the impact of recent lifestyle on the measurement of circulating irisin concentrations. One study considered cold exposure and controlled room temperature during the examination period [46].

**Table 1:** Characteristics of included studies

Study and year	Location	Study design	Sample size	Male/Female	Health status	Blood sample	ELISA kits	Analysis methods	NOS scores
Armandi A 2022 [56]	Italy	Cross-sectional	41	33/8	NAFLD	Plasma	Phoenix Pharmaceuticals	Spearman	8
Tentolouris A 2018 [46]	Greece	Cross-sectional	53	22/31	Healthy Controls	Plasma	Aviscera Bioscience	Univariate linear regression	7
Foda A. A. 2017a [57]	Egypt	Case–control	40	0/40	PCOS	Serum	BioVendor	Pearson	8
Foda A. A. 2017b [57]	Egypt	Case–control	40	0/40	healthy controls	Serum	BioVendor	Pearson	8
Foda A. A. 2019a [58]	Egypt	Case–control	50	0/50	PCOS	Serum	BioVendor	Pearson	6
Foda A. A. 2019b [58]	Egypt	Case–control	50	0/50	PCOS	Serum	BioVendor	Pearson	6
Jameel F 2015 [47]	Australia	Cross-sectional	49	28/21	healthy	Plasma	Adipogen	Spearman	6
Mehrabian S 2016a [59]	Iran	Case–control	26	0/26	Healthy Controls	Serum	Biovendor	Spearman	8
Mehrabian S 2016b [59]	Iran	Case–control	38	0/38	BF % > 30	Serum	Biovendor	Spearman	8
Rashid FA 2020 [60]	Iraq	Case–control	30	30/0	normal Weight group	Serum	Phoenix Pharmaceuticals	Pearson	6
Anastasilakis AD 2014 [33]	Greece	cross-sectional	122	61/61	healthy	Serum	Phoenix Pharmaceuticals	Spearman	8
Gouni-Berthold I 2013 [35]	Germany	Cross-sectional	72	72/0	mild hypercholesterolemia	Plasma	Aviscera Biosciences	Spearman or Pearson	7
Zhu H 2018 [61]	China	Case–control	40	30/10	healthy	Serum	USCN Life Science Inc	Spearman or Pearson	7
Liu JJ 2013 [19]	Singapore	Case–control	60	25/35	Non-diabetic controls	Plasma	USCN Life Science Inc	Pearson	8

*NOS* Newcastle–Ottawa Scale, *NAFLD* nonalcoholic fatty liver disease, *PCOS* polycystic ovary syndrome, *BF%* body fat percent

The association between irisin levels and at least one of the metabolic parameters was evaluated in 13 studies for glycaemic parameters and insulin resistance as well as in 11 studies for lipid metabolism parameters and anthropometric measurements. The statistical analyses to measure the correlation coefficients between irisin levels and metabolic parameters included Spearman correlation analysis in five studies, Pearson correlation analysis in six studies and simple linear regression in one study, and two studies did not state a specific method. Almost all studies reported unadjusted results, except for two studies, which provided adjusted data as well. The range of quality scores was from 6 to 8 with a median score of 8 for case–control studies and a median score of 7 for cross-sectional studies.

### Quality assessment

**Fig. 2:**
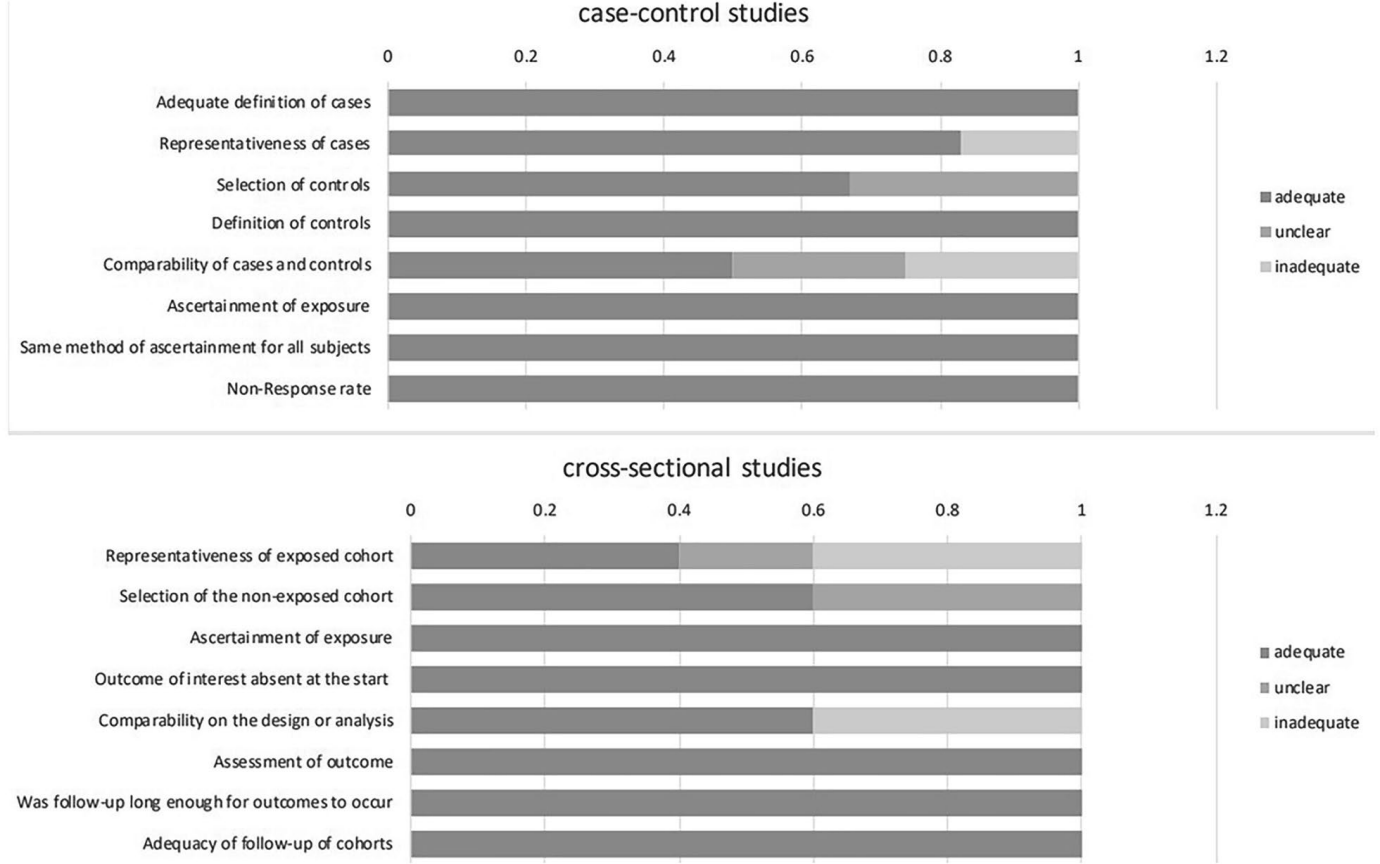
The proportion of included articles that reported each of the NOS items

Figure 2 illustrates the proportion of included articles that reported each of the NOS items. The majority of the included studies (n = 8) scored as of good quality (7–8 stars). The detailed quality assessment for each study is shown in Additional file 2.

### Association between irisin and metabolic parameters

#### Glucose metabolism, insulin levels and insulin resistance

**Fig. 3:**
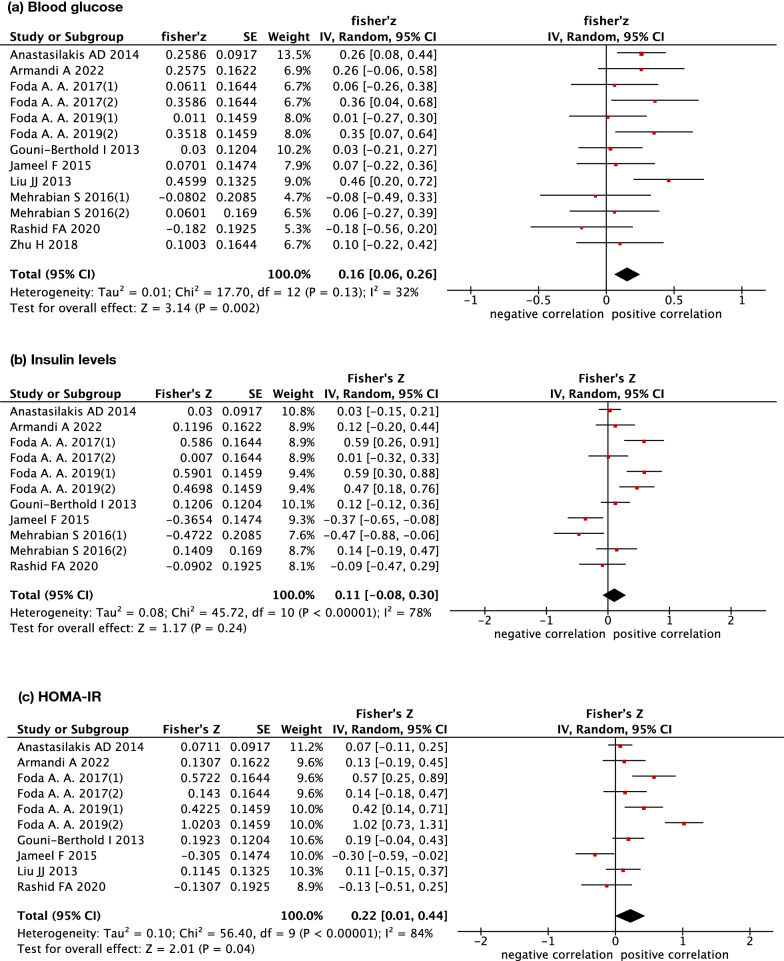
Forest plot detailing the Fisher's z transformation of correlation coefficients between irisin levels and fasting blood glucose, insulin levels and HOMA-IR

Of 14 studies calculating the correlation coefficient of circulating irisin with metabolic parameters, 13 studies reported the results of fasting blood glucose. Fisher's z transformation of correlation coefficients was calculated and imputed in the meta-analysis. The results (Fig. 3 and Table 2) showed that circulating irisin was positively and significantly correlated with fasting blood glucose (summary r = 0.159, 95% CI 0.060–0.254, p < 0.05). Moreover, sensitivity analysis suggested that the result was robust. The heterogeneity test demonstrated I^2^ = 32% (p > 0.05), indicating no significant heterogeneity among studies. The subgroup analyses by study design, blood sample, ELISA kits, male-to-female ratio, study location, metabolic status, study quality and whether overweight subjects were included in the study are shown in Additional file 3.

**Table 2:** The association between circulating irisin levels and metabolic parameters

	Summury r	95%CI
Glycemic profile		
FBG	0.159*	(0.060, 0.254)
fasting insulin	0.110	(−0.080, 0.291)
HOMA-IR	0.217*	(0.010, 0.414)
Lipid profile		
TG	0.070	(−0.080, 0.207)
TC	0.030	(−0.100, 0.149)
HDL-C	0.020	(−0.100, 0.139)
LDL-C	0.070	(−0.070, 0.188)
Anthropometric measurement		
BMI	0.149	(−0.000, 0.300)
WC	0.020	(−0.129, 0.178)
WHC	0.168*	(0.020, 0.310)

*CI* confidence interval, *FBG* fasting blood glucose, *HOMA-IR* homeostasis model assessment-insulin resistance, *TG* triglycerides, *TC* total cholesterol, *HDL-C* high-density lipoprotein cholesterol, *LDL-C* low-density lipoprotein cholesterol, *BMI* body mass index, *WC* waist circumference, *WHR* waist-to-hip ratio

Insulin levels and HOMA-IR were evaluated in 11 and 10 studies, respectively. The random-effect meta-analysis did not show a significant association between irisin and insulin levels (summary r = 0.110, 95% CI −0.080 to 0.291, p > 0.05). However, subgroup analysis by metabolic status showed that irisin was significantly correlated with insulin levels in metabolic disorder subjects (summary r = 0.327, 95% CI 0.149–0.485, p < 0.05). In contrast, a significant positive association was observed between irisin and HOMA-IR (summary r = 0.217, 95% CI 0.01–0.414, p < 0.05). Similar to insulin levels, the result only remained significant in metabolic disorder subjects (summary r = 0.438, 95% CI 0.149–0.653, p < 0.05) when the subgroup analysis was conducted (shown in Additional file 4).

#### Anthropometric measurement

**Fig. 4:**
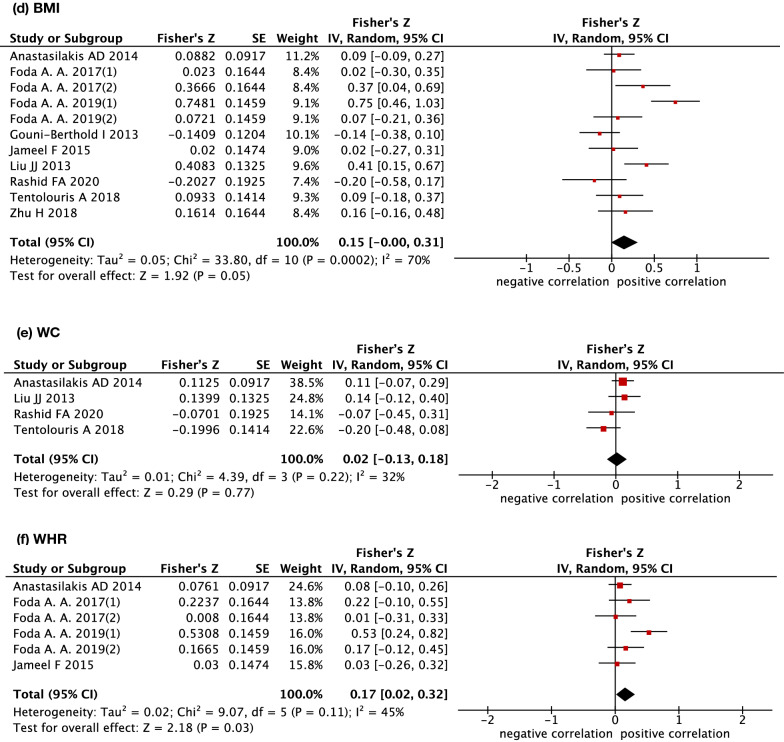
Forest plot detailing the Fisher's z transformation of correlation coefficients between irisin levels and BMI, WC and WHR

The association between irisin levels and anthropometric measurements is shown in Fig. 4 and Table 2. Eleven studies, 4 and 6 studies reported the results of BMI, WC and WHR, respectively. The random-effect meta-analysis did not show a significant association between irisin levels and BMI (summary r = 0.149, 95% CI −0.00 to 0.300, p = 0.05) with a medium-to-high level of heterogeneity across the included studies (I^2^ = 70%, p˂0.05). Subgroup analyses were also conducted to determine the correlation between irisin and BMI. The results showed that study design, ELISA kits, sex, study location and metabolic status were sources of heterogeneity as shown in Additional file 5. In addition, irisin levels were significantly and positively associated with BMI in the studies that included metabolically healthy individuals (summary *r* = 0.139; 95% CI 0.01–0.273), studies that included more female subjects (summary *r* = 0.282; 95% CI 0.060–0.470) and studies designed as case–control studies (summary *r* = 0.235; 95% CI 0.01–0.430) (shown in Additional file 5). In contrast, the pooled results showed that circulating irisin levels were significantly and positively associated with WHR (summary r = 0.168, 95% CI 0.020–0.310, p < 0.05). However, no significant correlation was detected between circulating irisin and WC (summary r = 0.020, 95% CI −0.129 to 0.178, p > 0.05). The heterogeneity was not significant in studies including the outcomes of WC and WHR.

#### Lipid profile

**Fig. 5:**
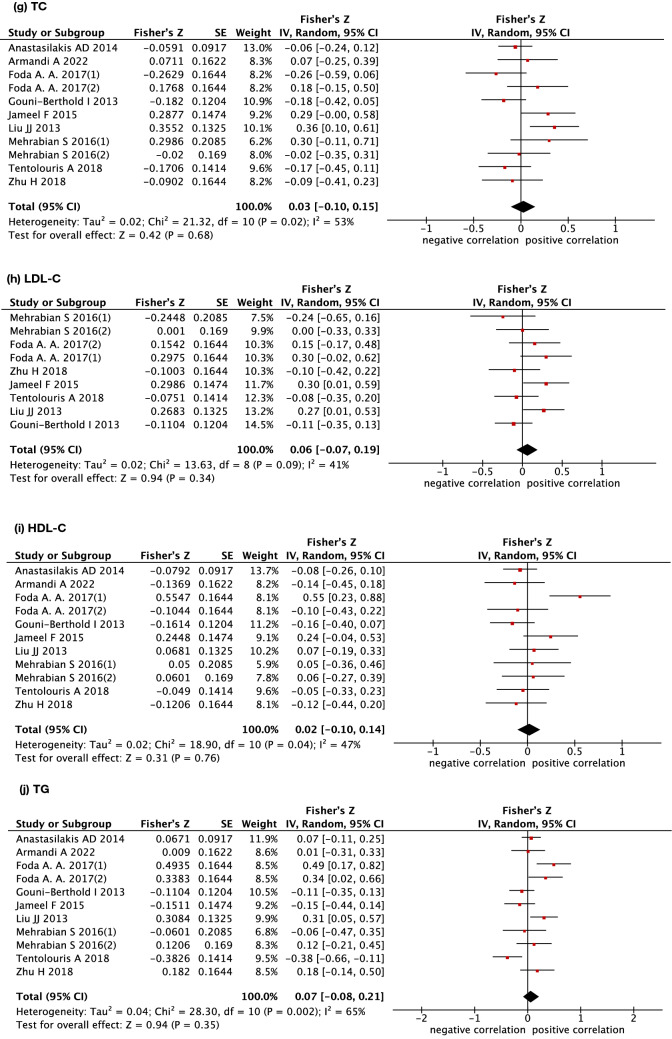
Forest plot detailing the Fisher's z transformation of correlation coefficients between irisin levels and lipid profile

The association between circulating irisin levels and lipid profiles was evaluated in 11 studies. Of note, 11 studies reported the outcome of TC, HDL-C and TG, and 9 studies reported the outcome of LDL-C. Overall, no significant correlation was found between irisin levels and TC (summary r = 0.030, 95% CI −0.100 to 0.149, p > 0.05), HDL-C (summary r = 0.020, 95% CI −0.100 to 0.139, p > 0.05), LDL-C (summary r = 0.070, 95% CI: −0.070 to 0.188, p > 0.05) or TG (summary r = 0.070, 95% CI −0.080 to 0.207, p > 0.05). In addition, there was a moderate-to-high level of statistical heterogeneity across studies for each outcome with I^2^ ranging from 50 to 70% (P < 0.05) (data are shown in Fig. 5 and Table 2).

### Publication bias

Funnel plots of included studies were presented in Additional file 6. (Due to the small number of studies included in several outcomes, the funnel plots were only performed in the outcomes of FBG, HOMA-IR, BMI, insulin, TC, HDL-C, and TG), and no significant publication bias was observed through visual evaluation of funnel plots. Egger’s test was also conducted, and the results were p = 0.1199 for FBG, p = 0.4201 for insulin, p = 0.9729 for HOMA-IR, p = 0.8866 for BMI, p = 0.4421 for TC, p = 0.3704 for HDL-C and p = 0.6461 for TG, demonstrating no evidence of publication bias in these metabolic parameters.

## Discussion

### Main findings

In the present study, we conducted a meta-analysis of 711 subjects in 14 studies to evaluate the association between irisin levels and metabolic parameters in nondiabetic, nonobese adults. We found that irisin levels were positively correlated with fasting blood glucose, HOMA-IR and WHR in this population. However, no significant association was detected between irisin levels and other metabolic parameters. To the best of our knowledge, this is the first meta-analysis to systematically evaluate the correlation between circulating irisin levels and metabolic parameters in a population without obesity or impaired glucose tolerance. Our findings provide a clue to understand the relationship between irisin and metabolic components in people with relatively normal metabolism.

### Interpretation

#### Glycaemic indices

Previous studies have indicated potential beneficial effects of irisin on glucose homeostasis and insulin resistance. However, human studies published to date [10, 14, 17, 19] have reported inconsistent results on the association between circulating irisin levels and glucose levels, insulin levels and insulin resistance in diabetic subjects. In contrast, in nondiabetic subjects, most studies have indicated that blood glucose is positively associated with irisin levels [10, 19, 21]. In the present meta-analysis, a significant positive correlation was also observed between irisin levels and fasting blood glucose when pooled across studies, and the estimate was robust across sensitivity analyses. Although the demonstrated correlation was relatively modest, our finding was in line with results from the literature. In addition, subgroup analyses indicated that the association between irisin levels and fasting blood glucose was likely to be influenced by the male-to-female ratio of the subjects, the method of irisin measurement, the study location and the NOS score of the study. Of note, irisin remained significantly associated with FBG only in studies that included overweight participants, whereas the correlation was not significant in studies that enrolled only normal weight subjects (BMI < 25 kg/m^2^), indicating the nonnegligible role of adiposity in the impact on circulating irisin levels, which may result from increased secretion from adiposity or resistance of irisin [9].

#### Insulin levels and insulin resistance

Studies have reported that irisin overexpression in mice fed a high-fat diet results in improved insulin sensitivity and glucose tolerance [17]. In humans, a cross-sectional study including 254 individuals with normal glucose tolerance has been conducted and found that irisin is positively correlated with circulating insulin levels and the homeostasis model assessment (HOMA)-β, even after adjustment for anthropometric and metabolic confounders [22]. A recent meta-analysis has revealed a significantly positive association between circulating irisin levels and insulin resistance in subjects with normal glucose tolerance [23]. In the present study, the pooled results did not show a significant association between circulating irisin and insulin levels. However, subgroup analysis by metabolic status demonstrated a significant and positive correlation between circulating irisin and insulin levels in studies that included patients with metabolic disorders (including three studies that enrolled PCOS patients, 1 study that enrolled NAFLD patients, one study that included individuals with mild hypercholesterolemia and one study that included people with BF% > 30). However, the results of studies enrolling metabolically healthy participants remained insignificant. In contrast, HOMA-IR was found to be significantly correlated with irisin levels, but subgroup analysis revealed a positive and significant correlation only in the metabolic disorder subgroup. These two outcomes combined indicated that insulin resistance and insulin levels are positively correlated with circulating levels of irisin under the condition of metabolic disorders but that the correlation is not obvious in metabolic healthy individuals. These findings were consistent with several studies, suggesting elevated circulating irisin levels in PCOS, metabolic syndrome, prediabetes and obese individuals in comparison with healthy controls [9, 11, 48, 49]. The increased secretion of irisin levels observed in metabolically abnormal individuals can be explained by increased adipose or muscle tissue in populations with metabolic disorders. Another possible explanation for the higher circulating irisin in metabolically abnormal patients is “irisin resistance”, a compensatory increase in circulating hormone levels to overcome resistance, similar to insulin resistance or leptin resistance [9, 50].

#### Anthropometric measurement

Numerous animal experiments and human research studies have focused on the potential association between irisin levels and obesity. Most studies have revealed a positive correlation of circulating irisin levels with BMI, weight, waist circumference waist-to-hip ratio and fat mass [9–15]. Furthermore, weight loss due to bariatric surgery lowers circulating irisin levels, which are restored after regaining the lost weight [11, 15], indicating the apparent link between adiposity and irisin. In contrast, one study including both normal-weight and overweight males did not detect any association between irisin and BMI [16]. Another study involving morbidly obese individuals has reported a negative association between irisin and BMI [17]. The contradictory results may be due to differences in health status and anthropometric parameters, especially the different degrees of obesity of the populations in these studies. It is well recognized that in healthy and normal weight populations, irisin in blood is mainly secreted by muscle cells, however, in obese individuals, the amount of irisin produced by adipose tissue is higher due to the increased amount of fat mass [15, 51], and it is plausible that the different amount of fat mass is a source of clinical heterogeneity in regard to the production of circulating irisin. Our results did not reveal a significant correlation between irisin and BMI in nondiabetic, nonobese subjects. Interestingly, subgroup analysis by male-to-female ratio revealed that the BMI of studies that included more female than male subjects was significantly associated with irisin, while studies with a male-to-female ratio > 1 showed a negative but insignificant correlation, suggesting a gender dimorphism of irisin in its relation to BMI. The results were confirmed by further subgroup analysis based on studies that included only male subjects and only female subjects. The sex difference in irisin has been explored in multiple studies [47, 52]. Females are suggested to have a higher irisin level than males after adjusting for potential confounders [33]. Similarly, another study has reported higher levels of irisin in healthy girls than in boys [52], suggesting sex differences in circulating irisin. A previously published study conducted in male and female subjects has reported sex discrepancies in the correlation of irisin with metabolic indices [47], and the author explained that the inconsistent results between males and females may be attributed to the different body compositions, such as fat distribution [53, 54], skeletal muscle mass and hormonal differences [10].

The pooled results also demonstrated a significant positive association between irisin and WHR as expected with robust results after conducting sensitivity analysis and low heterogeneity across studies. In contrast, no significant correlation was observed between irisin and WC, which may be due to insufficient studies included in regard to this outcome.

#### Lipid profile

Many studies have investigated the association between circulating irisin levels and lipid profiles with inconsistent findings. Animal experiments have suggested that the expression of FNDC5 or the administration of irisin promotes lipolysis and inhibits the synthesis of lipids [25–27]. In human studies, positive correlations between irisin levels and an unfavourable lipid profile have been reported [9, 28–30]. In contrast, a study that investigated obese subjects with nonalcoholic fatty liver disease (NAFLD) has suggested that lower blood triglycerides and transaminases are associated with a higher level of irisin [55], suggesting a favourable role of irisin in lipogenesis. Similarly, Anastasios et al. [46] found a negative and significant correlation between plasma irisin and fasting triglycerides, even after adjusting for potential confounders. However, in other studies, no significant association between irisin levels and lipid parameters has been observed [33–35]. In the present analysis, the lipid profile was evaluated in terms of the association with circulating irisin levels. The pooled results indicated that TC, HDL-C and LDL-C were not significantly correlated with irisin levels in the nondiabetic, nonobese population. The results remained largely unchanged after sensitivity analysis and subgroup analyses by study design, NOS score, ELISA kits, male-to-female ratio, metabolic status, study location, the method of irisin measurement and sample size of the studies. Notably, we did not observe a significant association between irisin and TG; however, by conducting subgroup analyses by study location, the direction of the correlation did not change but became significant and positive in studies conducted in Asia, indicating the possible impact of ethnicity on the correlation between irisin and TG.

### Limitation

The present study had several limitations. First, because we limited the population of our study to nonobese, nondiabetic subjects, the number of eligible studies with several results of interest was inadequate. Blood pressure, an important component of metabolic syndrome, was not included in the outcome due to the relatively small number of studies eligible for our meta-analysis. In addition, lifestyle, energy intake of participants and medication history were not considered for further subgroup analysis due to the limited number of studies included. Therefore, these factors that may affect the results need to be further clarified in future research. Second, although we excluded obese and diabetic populations from our meta-analysis, we did not exclude patients with other metabolic disorders, such as overweight, NAFLD, PCOS or dyslipidaemia. According to the literature, these disorders may also have some complex interactions with circulating irisin. However, we performed a subgroup analysis by metabolic status to analyse the clinical heterogeneity. Third, not all studies mentioned strict control of the diet and exercise of the participants. However, physical activity, especially the intensity and duration of the exercise or the type of training, can cause significant differences in irisin levels. Moreover, some results showed substantial heterogeneity across studies. Although we conducted a sensitivity analysis and subgroup analyses to explore the sources of heterogeneity, some remained unexplained, and meta-regression analysis was limited due to the insufficient number of studies included. Last, although several metabolic indices were found to be significantly associated with irisin levels, both the pooled results and individual studies showed a relatively modest correlation, suggesting that our results should be interpretated with caution.

## Conclusions

Our findings showed positive associations between irisin levels and several metabolic parameters in a relatively metabolically normal population. If irisin represents the link between the favourable influence of physical activity and improvement in metabolic status as well as if a clear association between irisin and certain metabolic diseases is confirmed by future studies, irisin may be a biomarker and therapeutic candidate in the treatment of these diseases. However, the regulation of irisin in humans and the role of irisin in glucose metabolism remain to be fully elucidated, and well-designed prospective studies are required. Additionally, the gender dimorphism of irisin observed in the present study indicated a need to investigate the association between irisin and metabolic syndrome based on different genders in future studies.

## Supplementary Information

**Additional file 1. **Search strategy and results.

**Additional file 2. **Methodological Quality of case–control and cross-sectional studies included in the meta-analysis.

**Additional file 3. **Summary of the subgroup analyses of the correlation between circulating irisin levels and fasting blood glucose.

**Additional file 4. **Summary of the subgroup analyses of the correlation between circulating irisin levels and HOMA-IR.

**Additional file 5. **Summary of the subgroup analyses of the correlation between circulating irisin levels and BMI.

**Additional file 6. **Funnel plots of included studies.

## Data Availability

Data are available from the authors upon reasonable request.
