# Supplementary material for: Association between irisin and metabolic parameters in nondiabetic, nonobese adults: a meta-analysis
Source: Diabetol Metab Syndr. 2022 Oct 21;14:152. doi: 10.1186/s13098-022-00922-w (PMC9585756; doi:10.1186/s13098-022-00922-w)
Supplement: Supplementary file 1 — Additional file 1. Search strategy and results. [file 13098_2022_922_MOESM1_ESM.docx]

Additional file 1: Search strategy and results.

| Databases | Search strategy | Results |
| --- | --- | --- |
| Pubmed | #1 irisin[Title/Abstract] OR myokine[Title/Abstract] | 2,037 |
|  | #2 "waist circumference"[MeSH Terms]OR "body mass index" [MeSH Terms] OR "body weight"[MeSH Terms]OR "waist to hip ratio"[MeSH Terms] OR "blood pressure"[MeSH Terms] OR “blood sugar” [MeSH Terms] OR "glycosylated hemoglobin" [MeSH Terms]OR “cholesterol, hdl”[MeSH Terms]OR “cholesterol, ldl” [MeSH Terms]OR triglycerides[MeSH Terms] OR triacylglycerol[MeSH Terms] | 1,066,896 |
|  | #3 "waist circumference"[Title/Abstract] OR "body mass index" [Title/Abstract] OR "body weight"[Title/Abstract]OR "waist to hip ratio"[Title/Abstract] OR "blood pressure"[Title/Abstract] OR glucose [Title/Abstract] OR “blood sugar” [Title/Abstract] OR insulin [Title/Abstract] OR HbA1c[Title/Abstract] OR "glycosylated hemoglobin" [Title/Abstract] OR HOMA-IR [Title/Abstract] OR "homeostasis model assessment-insulin resistance" [Title/Abstract] OR cholesterol [Title/Abstract] OR triglycerides[Title/Abstract] OR triacylglycerol [Title/Abstract] | 1,581,304 |
|  | #4 #2 OR #3 | 2,082,040 |
|  | #5 #1 AND #4 | 1,039 |
|  | Filters applied: Clinical Study, Clinical Trial, Comparative Study, Controlled Clinical Trial, Observational Study, Randomized Controlled Trial, Humans, Adult: 19+ years. | 82 |
|  | Search Date: 07/03/2022 |  |
|  |  |  |
| Embase | #1 'irisin'/exp | 1,913 |
|  | #2 irisin:ab,ti | 2,010 |
|  | #3 #1 OR #2 | 2,268 |
|  | #4 'waist circumference'/exp OR 'body mass'/exp OR 'body weight'/exp OR 'blood pressure'/exp OR 'glucose blood level'/exp OR 'insulin level'/exp OR 'insulin resistance'/exp OR 'hemoglobin a1c'/exp OR 'glycosylated hemoglobin'/exp OR 'homa index'/exp OR 'cholesterol'/exp OR 'triacylglycerol'/exp | 2,355,068 |
|  | #5 'waist circumference':ab,ti OR 'body mass index':ab,ti OR 'body weight':ab,ti OR 'blood pressure':ab,ti OR glucose:ab,ti OR insulin:ab,ti OR 'insulin resistance':ab,ti OR hba1c:ab,ti OR 'glycosylated hemoglobin':ab,ti OR 'homa ir':ab,ti OR cholesterol:ab,ti OR triacylglycerol:ab,ti OR triglycerides:ab,ti | 2,095,842 |
|  | #6 #4 OR #5 | 3,206,018 |
|  | #7 'case control study'/de OR 'clinical study'/de OR 'clinical trial'/de OR 'clinical trial topic'/de OR 'comparative study'/de OR 'controlled clinical trial'/de OR 'controlled study'/de OR 'cross sectional study'/de OR 'crossover procedure'/de OR 'double blind procedure'/de OR 'human'/de OR 'human experiment'/de OR 'intervention study'/de OR 'major clinical study'/de OR 'prospective study'/de OR 'randomized controlled trial'/de OR 'randomized controlled trial topic'/de OR 'retrospective study'/de | 28,302,217 |
|  | # 8 #3 AND #6 AND #7 | 631 |
|  | Search Date: 07/03/2022 |  |
|  |  |  |
| Cochrane | #1 (irisin):ti,ab,kw | 215 |
| library | #2 (myokine):ti,ab,kw | 85 |
|  | #3 #1 OR #2 | 256 |
|  | #4 MeSH descriptor: [Waist Circumference] explode all trees | 1,128 |
|  | #5 MeSH descriptor: [Body Mass Index] explode all trees | 10,782 |
|  | #6 MeSH descriptor: [Body Weight] explode all trees | 30,450 |
|  | #7 MeSH descriptor: [Blood Pressure] explode all trees | 28,387 |
|  | #8 MeSH descriptor: [Blood Glucose] explode all trees | 17,128 |
|  | #9 MeSH descriptor: [Insulin Resistance] explode all trees | 6,893 |
|  | #10 MeSH descriptor: [Glycated Hemoglobin A] explode all trees | 6,231 |
|  | #11 MeSH descriptor: [Cholesterol] explode all trees | 10,502 |
|  | #12 MeSH descriptor: [Triglycerides] explode all trees | 6,567 |
|  | #13 MeSH descriptor: [Triacylglycerol] explode all trees | 6,567 |
|  | #14 #4 OR #5 OR #6 OR #7 OR #8 OR #9 OR #10 OR #11 OR #12 OR #13 | 83,947 |
|  | #15 ("waist circumference"):ti,ab,kw OR ("body mass index"):ti,ab,kw OR ("body weight"):ti,ab,kw OR ("waist to hip ratio"):ti,ab,kw OR ("blood pressure"):ti,ab,kw OR (glucose):ti,ab,kw OR ("blood sugar"):ti,ab,kw OR ( insulin):ti,ab,kw OR (HbA1c):ti,ab,kw OR ("glycosylated hemoglobin"):ti,ab,kw OR (HOMA-IR):ti,ab,kw OR ("homeostasis model assessment-insulin resistance"):ti,ab,kw OR (cholesterol):ti,ab,kw OR (triglycerides):ti,ab,kw OR (triacylglycerol):ti,ab,kw | 255,193 |
|  | #16 #14 OR #15 | 264,327 |
|  | #17 #3 AND #16 | 168 |
|  | Select Page Tab“Trials” | 168 |
|  | Search Date: 07/03/2022 |  |
|  | Web of Science and ClinicalTrial.gov were also searched on the same day using website search engines. |  |
