# Supplementary material for: Association between irisin and metabolic parameters in nondiabetic, nonobese adults: a meta-analysis
Source: Diabetol Metab Syndr. 2022 Oct 21;14:152. doi: 10.1186/s13098-022-00922-w (PMC9585756; doi:10.1186/s13098-022-00922-w)
Supplement: Supplementary file 2 — Additional file 2. Methodological Quality of case–control and cross-sectional studies included in the meta-analysis. [file 13098_2022_922_MOESM2_ESM.docx]

| Additional file 2. Methodological Quality of case-control and cross-sectional studies included in the meta-analysis | | | | | | | | | |
| --- | --- | --- | --- | --- | --- | --- | --- | --- | --- |
| **case-control** |  |  |  |  |  |  |  |  |  |
| Study | Adequate definition of cases | Representativeness of cases | Selection of controls | Definition of controls | Comparability of cases and controls | Ascertainment of exposure | Same method of ascertainment for all subjects | Non-Response rate | Total quality score |
| Ashraf A. Foda 2017 | ※ | ※ | - | ※ | ※※ | ※ | ※ | ※ | 8 |
| Ashraf A. Foda 2019 | ※ | ※ | - | ※ | - | ※ | ※ | ※ | 6 |
| Sarvenaz Mehrabian2016 | ※ | ※ | ※ | ※ | ※ | ※ | ※ | ※ | 8 |
| Farah A. Rashid 2020 | ※ | - | * | ※ | - | ※ | ※ | ※ | 6 |
| Huijuan Zhu 2018 | ※ | ※ | ※ | ※ | ※※ | ※ | ※ | ※ | 7 |
| Liu JJ 2013 | ※ | ※ | ※ | ※ | ※ | ※ | ※ | ※ | 8 |
| **cross-sectional** |  |  |  |  |  |  |  |  |  |
| Study | Representativeness of exposed cohort | Selection of the non-exposed cohort | Ascertainment of exposure | Demonstration that outcome of interest was absent at the start of the study | Comparability of the cohorts on the design or analysis | Assessment of outcome | Was follow-up long enough for outcomes to occur | Adequacy of follow-up of cohorts | Total quality score |
| Angelo Armandi 2022 | ※ | ※ | ※ | ※ | ※ | ※ | ※ | ※ | 8 |
| Anastasios Tentolouris 2018 | ※ | - | ※ | ※ | ※ | ※ | ※ | ※ | 7 |
| Jameel F.2015 | - | - | ※ | ※ | ※ | ※ | ※ | ※ | 6 |
| Anastasilakis AD 2014 | - | ※ | ※ | ※ | ※※ | ※ | ※ | ※ | 8 |
| Gouni-Berthold I 2013 | - | ※ | ※ | ※ | ※ | ※ | ※ | ※ | 7 |

Newcastle–Ottawa Scale (NOS) was used to assess the methodological quality of each study. Eight items containing three aspects (selection, comparability and outcome or exposure) were scored for each study. The range of NOS was 0-9 stars.
