# Supplementary material for: Association between irisin and metabolic parameters in nondiabetic, nonobese adults: a meta-analysis
Source: Diabetol Metab Syndr. 2022 Oct 21;14:152. doi: 10.1186/s13098-022-00922-w (PMC9585756; doi:10.1186/s13098-022-00922-w)
Supplement: Supplementary file 3 — Additional file 3. Summary of the subgroup analyses of the correlation between circulating irisin levels and fasting blood glucose. [file 13098_2022_922_MOESM3_ESM.docx]

Additional file 3. Summary of the subgroup analyses of the correlation between circulating irisin levels and fasting blood glucose.

| Subgroups | Groups(n) | Summary r | 95%CI | P | Heterogeneity | |
| --- | --- | --- | --- | --- | --- | --- |
|  |  |  |  |  | I^2^(%) | p |
| Study design |  |  |  |  |  |  |
| Case-control | 9 | 0.149* | 0.010, 0.282 | 0.04 | 45 | 0.07 |
| Cross-sectional | 4 | 0.168* | 0.050, 0.282 | 0.006 | 1 | 0.39 |
| NOS score |  |  |  |  |  |  |
| ≥7 | 9 | 0.188* | 0.080, 0.291 | 0.0009 | 26 | 0.21 |
| ＜7 | 4 | 0.080 | -0.129, 0.282 | 0.45 | 46 | 0.14 |
| Blood sample of irisin |  |  |  |  |  |  |
| Plasma | 4 | 0.197 | -0.00, 0.388 | 0.05 | 55 | 0.08 |
| Serum | 9 | 0.139* | 0.020, 0.245 | 0.02 | 25 | 0.22 |
| Metabolic status |  |  |  |  |  |  |
| Metabolic disorders | 6 | 0.119* | 0.000,0.235 | 0.04 | 0 | 0.46 |
| Metabolically healthy | 7 | 0.168* | 0.020,0.319 | 0.03 | 50 | 0.06 |
| Male-to-female ratio |  |  |  |  |  |  |
| ＜1 | 7 | 0.188* | 0.040, 0.336 | 0.02 | 44 | 0.10 |
| ＞1 | 5 | 0.060 | -0.070, 0.197 | 0.35 | 0 | 0.53 |
| =1 | 1 | 0.254* | 0.080, 0.414 | 0.005 | - | - |
| Study location |  |  |  |  |  |  |
| Asia | 5 | 0.100 | -0.139, 0.319 | 0.42 | 60 | 0.04 |
| Europe | 3 | 0.178* | 0.040, 0.319 | 0.02 | 20 | 0.29 |
| Africa | 4 | 0.188* | 0.010, 0.363 | 0.04 | 32 | 0.22 |
| Australia | 1 | 0.070 | -0.217, 0.345 | 0.63 | - | - |
| ELISA kits |  |  |  |  |  |  |
| Phoenix Pharmaceuticals | 3 | 0.149 | -0.100, 0.371 | 0.23 | 55 | 0.11 |
| Other kits | 10 | 0.149* | 0.040, 0.264 | 0.008 | 31 | 0.16 |
| Included overweight subjects |  |  |  |  |  |  |
| Yes | 6 | 0.188* | 0.050, 0.310 | 0.008 | 43 | 0.12 |
| No | 7 | 0.119 | -0.030, 0.264 | 0.11 | 27 | 0.22 |

*p<0.05; CI: confidence interval; NOS: Newcastle–Ottawa Scale
