# Supplementary material for: Association between irisin and metabolic parameters in nondiabetic, nonobese adults: a meta-analysis
Source: Diabetol Metab Syndr. 2022 Oct 21;14:152. doi: 10.1186/s13098-022-00922-w (PMC9585756; doi:10.1186/s13098-022-00922-w)
Supplement: Supplementary file 4 — Additional file 4. Summary of the subgroup analyses of the correlation between circulating irisin levels and HOMA-IR. [file 13098_2022_922_MOESM4_ESM.docx]

Additional file 4. Summary of the subgroup analyses of the correlation between circulating irisin levels and HOMA-IR.

| Subgroups | Groups(n) | Summary r | 95%CI | | P | Heterogeneity | |
| --- | --- | --- | --- | --- | --- | --- | --- |
|  |  |  |  |  |  | I^2^(%) | p |
| Study design |  |  |  |  |  |  |  |
| Case-control | 6 | 0.8937* | 0.7779 | 0.9633 | 0.03 | 85 | ＜0.00001 |
| Cross-sectional | 4 | 0.7739 | 0.6911 | 0.8511 | 0.75 | 60 | 0.06 |
| NOS score |  |  |  |  |  |  |  |
| ≥7 | 6 | 0.1781* | 0.0500 | 0.3004 | 0.007 | 33 | 0.19 |
| ＜7 | 4 | 0.2543 | -0.3364 | 0.6963 | 0.41 | 94 | ＜0.00001 |
| Blood sample of irisin |  |  |  |  |  |  |  |
| Plasma | 4 | 0.0400 | -0.1781 | 0.2543 | 0.73 | 61 | 0.05 |
| Serum | 6 | 0.3364* | 0.0300 | 0.5915 | 0.03 | 87 | ＜0.00001 |
| Metabolic status |  |  |  |  |  |  |  |
| Metabolic disorders | 5 | 0.4120* | 0.1479 | 0.5737 | 0.004 | 84 | ＜0.0001 |
| Metabolically healthy | 5 | -0.0100 | -0.1577 | 0.1479 | 0.95 | 41 | 0.15 |
| Male-to-female ratio |  |  |  |  |  |  |  |
| ＜1 | 5 | 0.4219* | 0.1194 | 0.6584 | 0.008 | 84 | ＜0.0001 |
| ＞1 | 4 | -0.0200 | -0.2543 | 0.2260 | 0.89 | 62 | 0.05 |
| =1 | 1 | 0.0699 | -0.1096 | 0.2449 | 0.44 | - | - |
| Study location |  |  |  |  |  |  |  |
| Asia | 2 | 0.0300 | -0.1974 | 0.2543 | 0.78 | 9 | 0.29 |
| Europe | 3 | 0.1194 | -0.0100 | 0.2449 | 0.08 | 0 | 0.72 |
| Africa | 4 | 0.4930* | 0.1781 | 0.7211 | 0.003 | 83 | 0.0006 |
| Australia | 1 | -0.2913* | -0.5299 | -0.0200 | 0.04 | - | - |
| ELISA kits |  |  |  |  |  |  |  |
| Phoenix Pharmaceuticals | 3 | 0.0500 | -0.0898 | 0.1974 | 0.47 | 0 | 0.55 |
| Other kits | 7 | 0.3004 | 0.000 | 0.5441 | 0.05 | 88 | ＜0.00001 |
| Included overweight subjects |  |  |  |  |  |  |  |
| Yes | 6 | 0.1096 | -0.0599 | 0.2636 | 0.22 | 62 | 0.02 |
| No | 4 | 0.3885 | -0.0898 | 0.7211 | 0.11 | 89 | ＜0.00001 |

*p<0.05; HOMA-IR: homeostasis model assessment-insulin resistance; CI: confidence interval; NOS: Newcastle–Ottawa Scale
