# Supplementary material for: Association between irisin and metabolic parameters in nondiabetic, nonobese adults: a meta-analysis
Source: Diabetol Metab Syndr. 2022 Oct 21;14:152. doi: 10.1186/s13098-022-00922-w (PMC9585756; doi:10.1186/s13098-022-00922-w)
Supplement: Supplementary file 5 — Additional file 5. Summary of the subgroup analyses of the correlation between circulating irisin levels and BMI. [file 13098_2022_922_MOESM5_ESM.docx]

Additional file 5. Summary of the subgroup analyses of the correlation between circulating irisin levels and BMI.

| Subgroups | Groups(n) | Summary r | 95%CI | | P | Heterogeneity | |
| --- | --- | --- | --- | --- | --- | --- | --- |
|  |  |  |  |  |  | I^2^(%) | p |
| Study design |  |  |  |  |  |  |  |
| Case-control | 7 | 0.235* | 0.01 | 0.430 | 0.04 | 74 | 0.0009 |
| Cross-sectional | 4 | 0.020 | -0.090 | 0.139 | 0.71 | 0 | 0.46 |
| NOS score |  |  |  |  |  |  |  |
| ≥7 | 7 | 0.129 | -0.01 | 0.273 | 0.07 | 51 | 0.06 |
| ＜7 | 4 | 0.168 | -0.236 | 0.515 | 0.41 | 86 | 0.0001 |
| Blood sample of irisin |  |  |  |  |  |  |  |
| Plasma | 4 | 0.0898 | -0.139 | 0.319 | 0.44 | 69 | 0.02 |
| Serum | 7 | 0.188 | -0.030 | 0.380 | 0.09 | 74 | 0.0008 |
| Metabolic status |  |  |  |  |  |  |  |
| Metabolic disorders | 4 | 0.168 | -0.217 | 0.515 | 0.39 | 87 | ＜0.0001 |
| Metabolically healthy | 7 | 0.139* | 0.01 | 0.273 | 0.04 | 42 | 0.11 |
| Male-to-female ratio |  |  |  |  |  |  |  |
| ＜1 | 6 | 0.282* | 0.060 | 0.470 | 0.01 | 72 | 0.003 |
| Only females |  |  |  |  |  |  |  |
| ＞1 | 4 | -0.050 | -0.188 | 0.100 | 0.54 | 2 | 0.38 |
| Only males |  |  |  |  |  |  |  |
| =1 | 1 | 0.090 | -0.090 | 0.264 | 0.34 | - | - |
| Study location |  |  |  |  |  |  |  |
| Asia | 3 | 0.139 | -0.197 | 0.446 | 0.41 | 71 | 0.03 |
| Europe | 3 | 0.020 | -0.129 | 0.168 | 0.80 | 23 | 0.27 |
| Africa | 4 | 0.300 | -0.030 | 0.565 | 0.08 | 80 | 0.002 |
| Australia | 1 | 0.020 | -0.263 | 0.300 | 0.89 | - | - |
| ELISA kits |  |  |  |  |  |  |  |
| Phoenix Pharmaceuticals | 2 | -0.01 | -0.273 | 0.254 | 0.95 | 46 | 0.17 |
| Other kits | 9 | 0.188* | 0.01 | 0.363 | 0.04 | 73 | 0.0003 |
| Included overweight subjects |  |  |  |  |  |  |  |
| Yes | 6 | 0.197 | -0.040 | 0.414 | 0.11 | 82 | ＜0.0001 |
| No | 5 | 0.10 | -0.070 | 0.264 | 0.27 | 27 | 0.24 |

*p<0.05; BMI: body mass index; CI: confidence interval; NOS: Newcastle–Ottawa Scale
