# Supplementary figures and images for: Association between irisin and metabolic parameters in nondiabetic, nonobese adults: a meta-analysis
Source: Diabetol Metab Syndr. 2022 Oct 21;14:152. doi: 10.1186/s13098-022-00922-w (PMC9585756; doi:10.1186/s13098-022-00922-w)

(a)

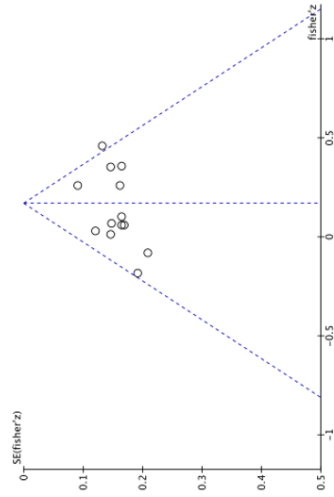

(b)

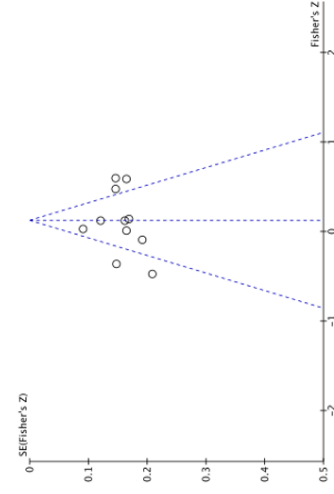

(e)

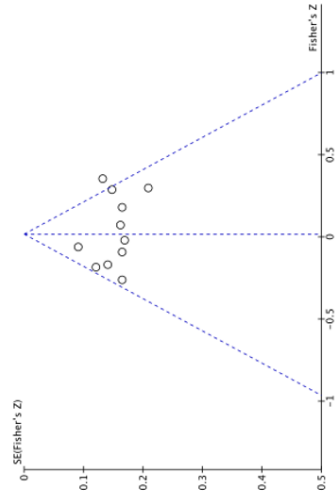

(f)

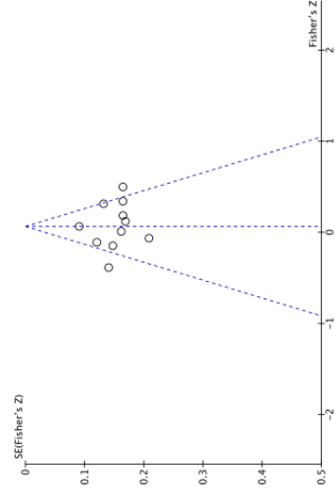

(c)

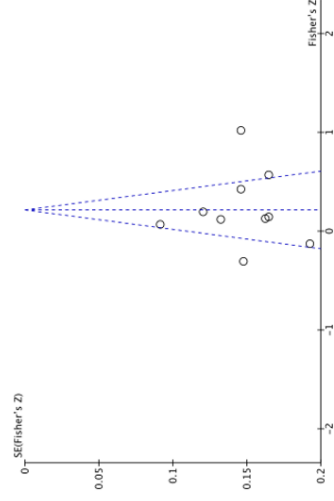

(g)

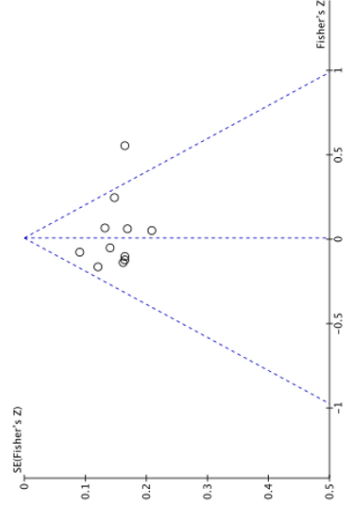

(d)

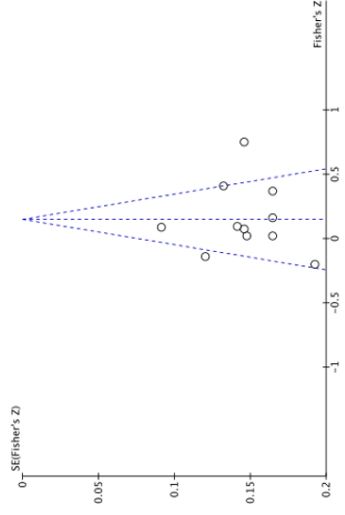

Supplement: Supplementary file 6 — Additional file 6. Funnel plots of included studies. [file 13098_2022_922_MOESM6_ESM.pdf]
